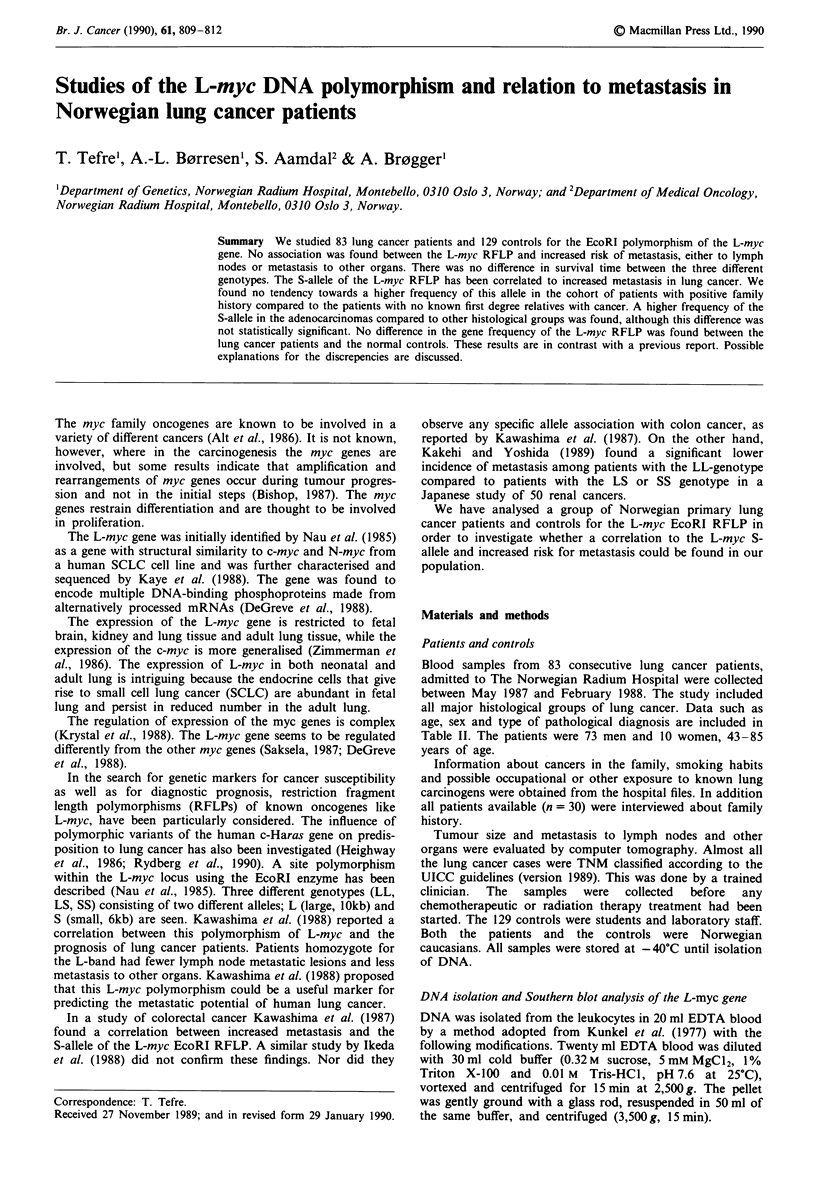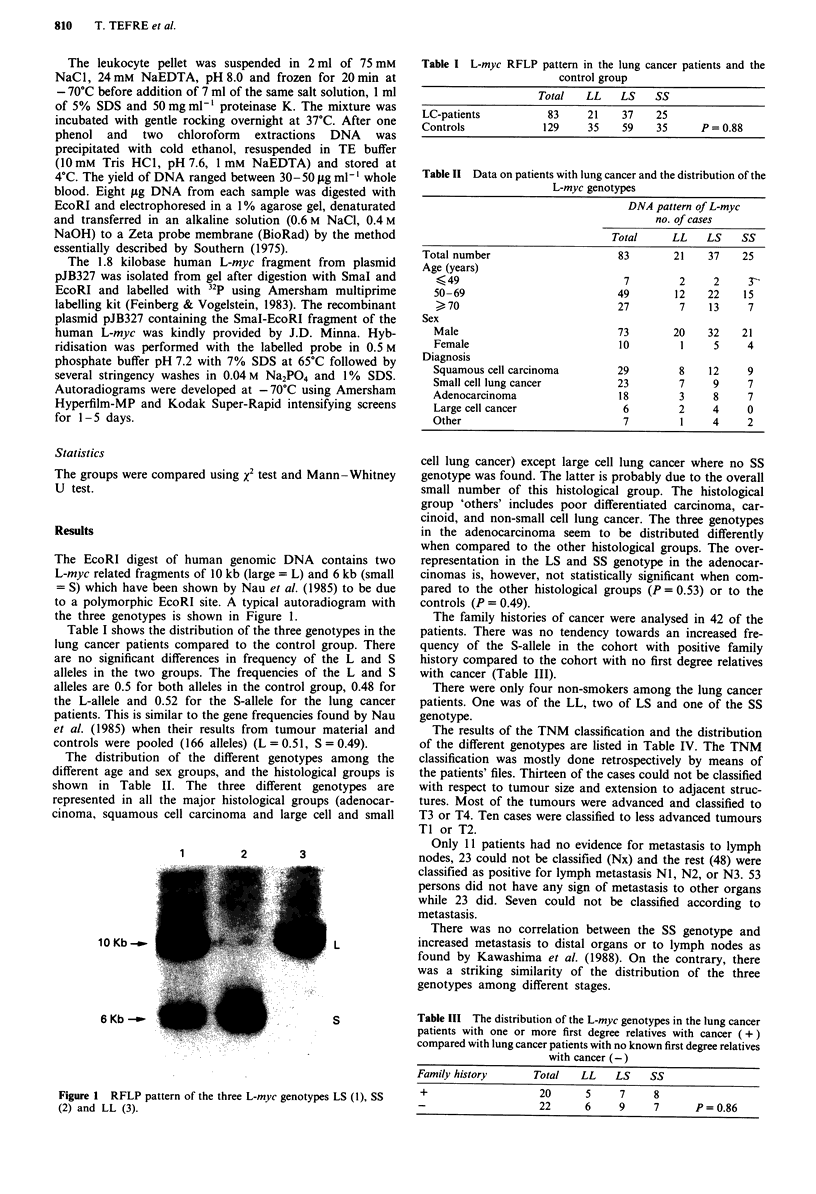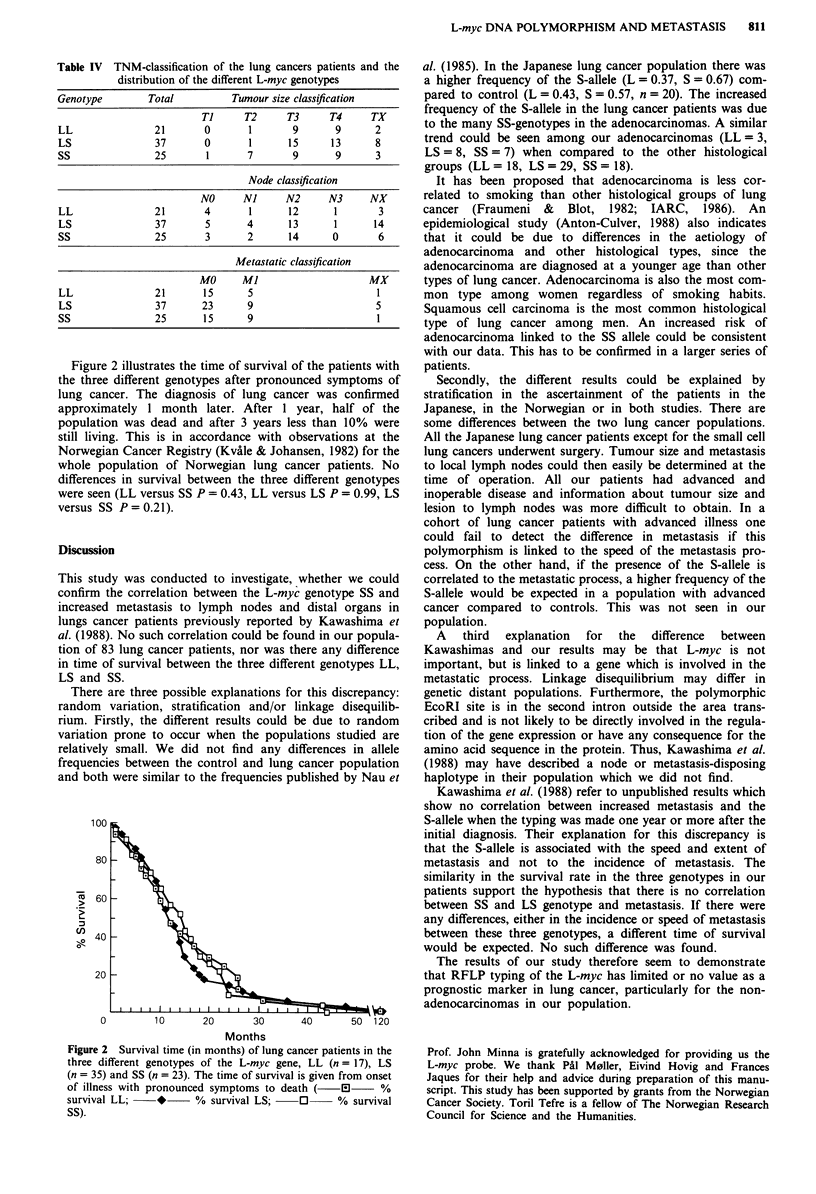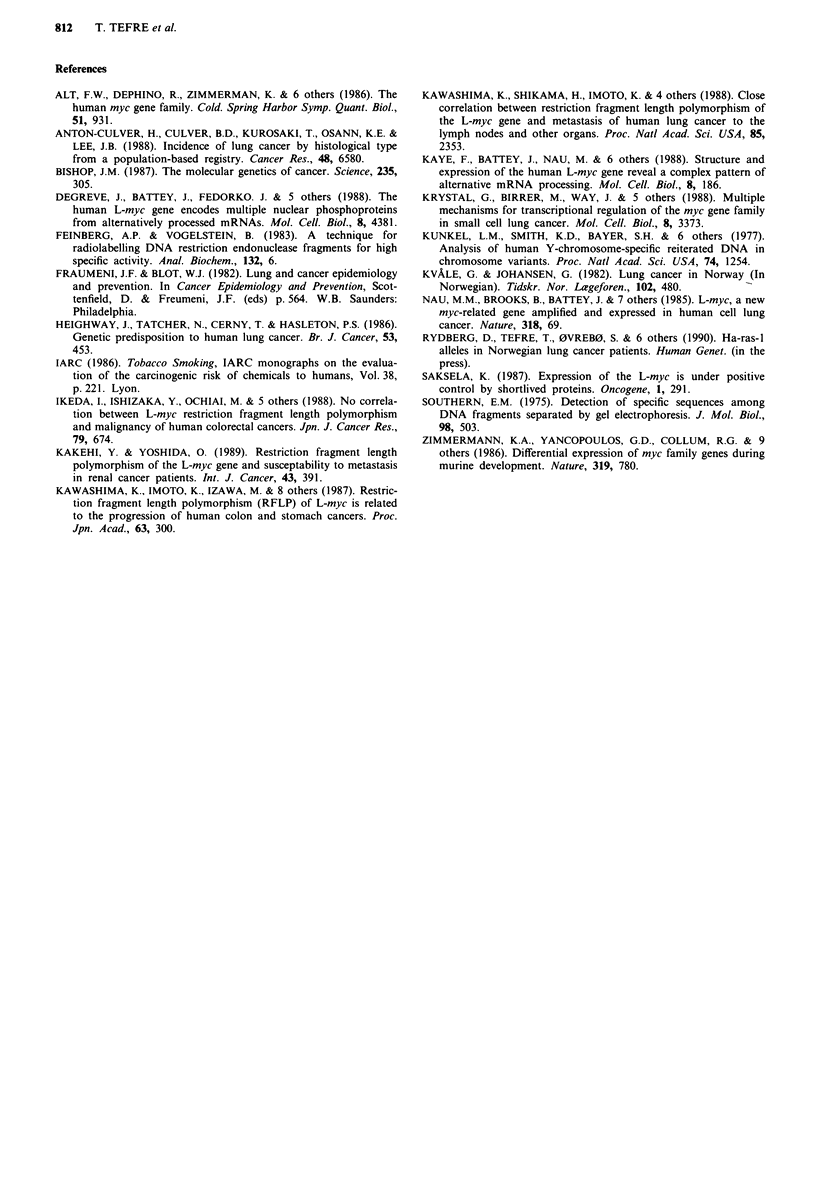# Studies of the L-myc DNA polymorphism and relation to metastasis in Norwegian lung cancer patients.

**DOI:** 10.1038/bjc.1990.182

**Published:** 1990-06

**Authors:** T. Tefre, A. L. Børresen, S. Aamdal, A. Brøgger

**Affiliations:** Department of Genetics, Norwegian Radium Hospital, Oslo, Norway.

## Abstract

**Images:**


Br. J. Cancer (1990), 61, 809-812 © Macmillan Press Ltd., 1990

### Studies of the L-*myc* DNA polymorphism and relation to metastasis in Norwegian lung cancer patients

T. Tefre[1], A.-L. Børresen[1], S. Aamdal[2] & A. Brøgger[1]

[1]*Department of Genetics, Norwegian Radium Hospital, Montebello, 0310 Oslo 3, Norway; and* [2]*Department of Medical Oncology, Norwegian Radium Hospital, Montebello, 0310 Oslo 3, Norway.*

**Summary** We studied 83 lung cancer patients and 129 controls for the EcoRI polymorphism of the L-*myc* gene. No association was found between the L-*myc* RFLP and increased risk of metastasis, either to lymph nodes or metastasis to other organs. There was no difference in survival time between the three different genotypes. The S-allele of the L-*myc* RFLP has been correlated to increased metastasis in lung cancer. We found no tendency towards a higher frequency of this allele in the cohort of patients with positive family history compared to the patients with no known first degree relatives with cancer. A higher frequency of the S-allele in the adenocarcinomas compared to other histological groups was found, although this difference was not statistically significant. No difference in the gene frequency of the L-*myc* RFLP was found between the lung cancer patients and the normal controls. These results are in contrast with a previous report. Possible explanations for the discrepencies are discussed.

The *myc* family oncogenes are known to be involved in a variety of different cancers (Alt *et al.*, 1986). It is not known, however, where in the carcinogenesis the *myc* genes are involved, but some results indicate that amplification and rearrangements of *myc* genes occur during tumour progression and not in the initial steps (Bishop, 1987). The *myc* genes restrain differentiation and are thought to be involved in proliferation.

The L-*myc* gene was initially identified by Nau *et al.* (1985) as a gene with structural similarity to c-*myc* and N-*myc* from a human SCLC cell line and was further characterised and sequenced by Kaye *et al.* (1988). The gene was found to encode multiple DNA-binding phosphoproteins made from alternatively processed mRNAs (DeGreve *et al.*, 1988).

The expression of the L-*myc* gene is restricted to fetal brain, kidney and lung tissue and adult lung tissue, while the expression of the c-*myc* is more generalised (Zimmerman *et al.*, 1986). The expression of L-*myc* in both neonatal and adult lung is intriguing because the endocrine cells that give rise to small cell lung cancer (SCLC) are abundant in fetal lung and persist in reduced number in the adult lung.

The regulation of expression of the myc genes is complex (Krystal *et al.*, 1988). The L-*myc* gene seems to be regulated differently from the other *myc* genes (Saksela, 1987; DeGreve *et al.*, 1988).

In the search for genetic markers for cancer susceptibility as well as for diagnostic prognosis, restriction fragment length polymorphisms (RFLPs) of known oncogenes like L-*myc*, have been particularly considered. The influence of polymorphic variants of the human c-Ha*ras* gene on predisposition to lung cancer has also been investigated (Heighway *et al.*, 1986; Rydberg *et al.*, 1990). A site polymorphism within the L-*myc* locus using the EcoRI enzyme has been described (Nau *et al.*, 1985). Three different genotypes (LL, LS, SS) consisting of two different alleles; L (large, 10kb) and S (small, 6kb) are seen. Kawashima *et al.* (1988) reported a correlation between this polymorphism of L-*myc* and the prognosis of lung cancer patients. Patients homozygote for the L-band had fewer lymph node metastatic lesions and less metastasis to other organs. Kawashima *et al.* (1988) proposed that this L-*myc* polymorphism could be a useful marker for predicting the metastatic potential of human lung cancer.

In a study of colorectal cancer Kawashima *et al.* (1987) found a correlation between increased metastasis and the S-allele of the L-*myc* EcoRI RFLP. A similar study by Ikeda *et al.* (1988) did not confirm these findings. Nor did they observe any specific allele association with colon cancer, as reported by Kawashima *et al.* (1987). On the other hand, Kakehi and Yoshida (1989) found a significant lower incidence of metastasis among patients with the LL-genotype compared to patients with the LS or SS genotype in a Japanese study of 50 renal cancers.

We have analysed a group of Norwegian primary lung cancer patients and controls for the L-*myc* EcoRI RFLP in order to investigate whether a correlation to the L-*myc* S-allele and increased risk for metastasis could be found in our population.

### Materials and methods

### *Patients and controls*

Blood samples from 83 consecutive lung cancer patients, admitted to The Norwegian Radium Hospital were collected between May 1987 and February 1988. The study included all major histological groups of lung cancer. Data such as age, sex and type of pathological diagnosis are included in Table II. The patients were 73 men and 10 women, 43-85 years of age.

Information about cancers in the family, smoking habits and possible occupational or other exposure to known lung carcinogens were obtained from the hospital files. In addition all patients available ($n = 30$) were interviewed about family history.

Tumour size and metastasis to lymph nodes and other organs were evaluated by computer tomography. Almost all the lung cancer cases were TNM classified according to the UICC guidelines (version 1989). This was done by a trained clinician. The samples were collected before any chemotherapeutic or radiation therapy treatment had been started. The 129 controls were students and laboratory staff. Both the patients and the controls were Norwegian caucasians. All samples were stored at $-40°C$ until isolation of DNA.

### *DNA isolation and Southern blot analysis of the L-*myc *gene*

DNA was isolated from the leukocytes in 20 ml EDTA blood by a method adopted from Kunkel *et al.* (1977) with the following modifications. Twenty ml EDTA blood was diluted with 30 ml cold buffer (0.32 M sucrose, 5 mM $MgCl_2$, 1% Triton X-100 and 0.01 M Tris-HCl, pH 7.6 at 25°C), vortexed and centrifuged for 15 min at 2,500 *g*. The pellet was gently ground with a glass rod, resuspended in 50 ml of the same buffer, and centrifuged (3,500 *g*, 15 min).

Correspondence: T. Tefre.
Received 27 November 1989; and in revised form 29 January 1990.

The leukocyte pellet was suspended in 2 ml of 75 mM NaCl, 24 mM NaEDTA, pH 8.0 and frozen for 20 min at −70°C before addition of 7 ml of the same salt solution, 1 ml of 5% SDS and 50 mg ml$^{-1}$ proteinase K. The mixture was incubated with gentle rocking overnight at 37°C. After one phenol and two chloroform extractions DNA was precipitated with cold ethanol, resuspended in TE buffer (10 mM Tris HCl, pH 7.6, 1 mM NaEDTA) and stored at 4°C. The yield of DNA ranged between 30–50 μg ml$^{-1}$ whole blood. Eight μg DNA from each sample was digested with EcoRI and electrophoresed in a 1% agarose gel, denaturated and transferred in an alkaline solution (0.6 M NaCl, 0.4 M NaOH) to a Zeta probe membrane (BioRad) by the method essentially described by Southern (1975).

The 1.8 kilobase human L-*myc* fragment from plasmid pJB327 was isolated from gel after digestion with SmaI and EcoRI and labelled with $^{32}P$ using Amersham multiprime labelling kit (Feinberg & Vogelstein, 1983). The recombinant plasmid pJB327 containing the SmaI-EcoRI fragment of the human L-*myc* was kindly provided by J.D. Minna. Hybridisation was performed with the labelled probe in 0.5 M phosphate buffer pH 7.2 with 7% SDS at 65°C followed by several stringency washes in 0.04 M $Na_2PO_4$ and 1% SDS. Autoradiograms were developed at −70°C using Amersham Hyperfilm-MP and Kodak Super-Rapid intensifying screens for 1–5 days.

*Statistics*

The groups were compared using $\chi^2$ test and Mann–Whitney U test.

### Results

The EcoRI digest of human genomic DNA contains two L-*myc* related fragments of 10 kb (large = L) and 6 kb (small = S) which have been shown by Nau *et al.* (1985) to be due to a polymorphic EcoRI site. A typical autoradiogram with the three genotypes is shown in Figure 1.

Table I shows the distribution of the three genotypes in the lung cancer patients compared to the control group. There are no significant differences in frequency of the L and S alleles in the two groups. The frequencies of the L and S alleles are 0.5 for both alleles in the control group, 0.48 for the L-allele and 0.52 for the S-allele for the lung cancer patients. This is similar to the gene frequencies found by Nau *et al.* (1985) when their results from tumour material and controls were pooled (166 alleles) (L = 0.51, S = 0.49).

The distribution of the different genotypes among the different age and sex groups, and the histological groups is shown in Table II. The three different genotypes are represented in all the major histological groups (adenocarcinoma, squamous cell carcinoma and large cell and small

**Figure 1** RFLP pattern of the three L-*myc* genotypes LS (1), SS (2) and LL (3).

**Table I** L-*myc* RFLP pattern in the lung cancer patients and the control group

| | *Total* | *LL* | *LS* | *SS* | |
|---|---|---|---|---|---|
| LC-patients | 83 | 21 | 37 | 25 | |
| Controls | 129 | 35 | 59 | 35 | $P = 0.88$ |

**Table II** Data on patients with lung cancer and the distribution of the L-*myc* genotypes

| | *DNA pattern of L-myc no. of cases* | | | |
|---|---|---|---|---|
| | *Total* | *LL* | *LS* | *SS* |
| Total number | 83 | 21 | 37 | 25 |
| Age (years) | | | | |
| ≤49 | 7 | 2 | 2 | 3 |
| 50–69 | 49 | 12 | 22 | 15 |
| ≥70 | 27 | 7 | 13 | 7 |
| Sex | | | | |
| Male | 73 | 20 | 32 | 21 |
| Female | 10 | 1 | 5 | 4 |
| Diagnosis | | | | |
| Squamous cell carcinoma | 29 | 8 | 12 | 9 |
| Small cell lung cancer | 23 | 7 | 9 | 7 |
| Adenocarcinoma | 18 | 3 | 8 | 7 |
| Large cell cancer | 6 | 2 | 4 | 0 |
| Other | 7 | 1 | 4 | 2 |

cell lung cancer) except large cell lung cancer where no SS genotype was found. The latter is probably due to the overall small number of this histological group. The histological group ‘others’ includes poor differentiated carcinoma, carcinoid, and non-small cell lung cancer. The three genotypes in the adenocarcinoma seem to be distributed differently when compared to the other histological groups. The over-representation in the LS and SS genotype in the adenocarcinomas is, however, not statistically significant when compared to the other histological groups ($P = 0.53$) or to the controls ($P = 0.49$).

The family histories of cancer were analysed in 42 of the patients. There was no tendency towards an increased frequency of the S-allele in the cohort with positive family history compared to the cohort with no first degree relatives with cancer (Table III).

There were only four non-smokers among the lung cancer patients. One was of the LL, two of LS and one of the SS genotype.

The results of the TNM classification and the distribution of the different genotypes are listed in Table IV. The TNM classification was mostly done retrospectively by means of the patients' files. Thirteen of the cases could not be classified with respect to tumour size and extension to adjacent structures. Most of the tumours were advanced and classified to T3 or T4. Ten cases were classified to less advanced tumours T1 or T2.

Only 11 patients had no evidence for metastasis to lymph nodes, 23 could not be classified (Nx) and the rest (48) were classified as positive for lymph metastasis N1, N2, or N3. 53 persons did not have any sign of metastasis to other organs while 23 did. Seven could not be classified according to metastasis.

There was no correlation between the SS genotype and increased metastasis to distal organs or to lymph nodes as found by Kawashima *et al.* (1988). On the contrary, there was a striking similarity of the distribution of the three genotypes among different stages.

**Table III** The distribution of the L-*myc* genotypes in the lung cancer patients with one or more first degree relatives with cancer (+) compared with lung cancer patients with no known first degree relatives with cancer (−)

| *Family history* | *Total* | *LL* | *LS* | *SS* | |
|---|---|---|---|---|---|
| + | 20 | 5 | 7 | 8 | |
| − | 22 | 6 | 9 | 7 | $P = 0.86$ |

**Table IV** TNM-classification of the lung cancers patients and the distribution of the different L-*myc* genotypes

| *Genotype* | *Total* | *Tumour size classification* | | | | |
|---|---|---|---|---|---|---|
| | | *T1* | *T2* | *T3* | *T4* | *TX* |
| LL | 21 | 0 | 1 | 9 | 9 | 2 |
| LS | 37 | 0 | 1 | 15 | 13 | 8 |
| SS | 25 | 1 | 7 | 9 | 9 | 3 |
| | | *Node classification* | | | | |
| | | *N0* | *N1* | *N2* | *N3* | *NX* |
| LL | 21 | 4 | 1 | 12 | 1 | 3 |
| LS | 37 | 5 | 4 | 13 | 1 | 14 |
| SS | 25 | 3 | 2 | 14 | 0 | 6 |
| | | *Metastatic classification* | | | | |
| | | *M0* | *M1* | | | *MX* |
| LL | 21 | 15 | 5 | | | 1 |
| LS | 37 | 23 | 9 | | | 5 |
| SS | 25 | 15 | 9 | | | 1 |

Figure 2 illustrates the time of survival of the patients with the three different genotypes after pronounced symptoms of lung cancer. The diagnosis of lung cancer was confirmed approximately 1 month later. After 1 year, half of the population was dead and after 3 years less than 10% were still living. This is in accordance with observations at the Norwegian Cancer Registry (Kvåle & Johansen, 1982) for the whole population of Norwegian lung cancer patients. No differences in survival between the three different genotypes were seen (LL versus SS $P = 0.43$, LL versus LS $P = 0.99$, LS versus SS $P = 0.21$).

### Discussion

This study was conducted to investigate, whether we could confirm the correlation between the L-*myc* genotype SS and increased metastasis to lymph nodes and distal organs in lungs cancer patients previously reported by Kawashima *et al.* (1988). No such correlation could be found in our population of 83 lung cancer patients, nor was there any difference in time of survival between the three different genotypes LL, LS and SS.

There are three possible explanations for this discrepancy: random variation, stratification and/or linkage disequilibrium. Firstly, the different results could be due to random variation prone to occur when the populations studied are relatively small. We did not find any differences in allele frequencies between the control and lung cancer population and both were similar to the frequencies published by Nau *et al.* (1985). In the Japanese lung cancer population there was a higher frequency of the S-allele (L = 0.37, S = 0.67) compared to control (L = 0.43, S = 0.57, $n = 20$). The increased frequency of the S-allele in the lung cancer patients was due to the many SS-genotypes in the adenocarcinomas. A similar trend could be seen among our adenocarcinomas (LL = 3, LS = 8, SS = 7) when compared to the other histological groups (LL = 18, LS = 29, SS = 18).

**Figure 2** Survival time (in months) of lung cancer patients in the three different genotypes of the L-*myc* gene, LL ($n = 17$), LS ($n = 35$) and SS ($n = 23$). The time of survival is given from onset of illness with pronounced symptoms to death (——⊡—— % survival LL; ——◆—— % survival LS; ——□—— % survival SS).

It has been proposed that adenocarcinoma is less correlated to smoking than other histological groups of lung cancer (Fraumeni & Blot, 1982; IARC, 1986). An epidemiological study (Anton-Culver, 1988) also indicates that it could be due to differences in the aetiology of adenocarcinoma and other histological types, since the adenocarcinoma are diagnosed at a younger age than other types of lung cancer. Adenocarcinoma is also the most common type among women regardless of smoking habits. Squamous cell carcinoma is the most common histological type of lung cancer among men. An increased risk of adenocarcinoma linked to the SS allele could be consistent with our data. This has to be confirmed in a larger series of patients.

Secondly, the different results could be explained by stratification in the ascertainment of the patients in the Japanese, in the Norwegian or in both studies. There are some differences between the two lung cancer populations. All the Japanese lung cancer patients except for the small cell lung cancers underwent surgery. Tumour size and metastasis to local lymph nodes could then easily be determined at the time of operation. All our patients had advanced and inoperable disease and information about tumour size and lesion to lymph nodes was more difficult to obtain. In a cohort of lung cancer patients with advanced illness one could fail to detect the difference in metastasis if this polymorphism is linked to the speed of the metastasis process. On the other hand, if the presence of the S-allele is correlated to the metastatic process, a higher frequency of the S-allele would be expected in a population with advanced cancer compared to controls. This was not seen in our population.

A third explanation for the difference between Kawashimas and our results may be that L-*myc* is not important, but is linked to a gene which is involved in the metastatic process. Linkage disequilibrium may differ in genetic distant populations. Furthermore, the polymorphic EcoRI site is in the second intron outside the area transcribed and is not likely to be directly involved in the regulation of the gene expression or have any consequence for the amino acid sequence in the protein. Thus, Kawashima *et al.* (1988) may have described a node or metastasis-disposing haplotype in their population which we did not find.

Kawashima *et al.* (1988) refer to unpublished results which show no correlation between increased metastasis and the S-allele when the typing was made one year or more after the initial diagnosis. Their explanation for this discrepancy is that the S-allele is associated with the speed and extent of metastasis and not to the incidence of metastasis. The similarity in the survival rate in the three genotypes in our patients support the hypothesis that there is no correlation between SS and LS genotype and metastasis. If there were any differences, either in the incidence or speed of metastasis between these three genotypes, a different time of survival would be expected. No such difference was found.

The results of our study therefore seem to demonstrate that RFLP typing of the L-*myc* has limited or no value as a prognostic marker in lung cancer, particularly for the non-adenocarcinomas in our population.

Prof. John Minna is gratefully acknowledged for providing us the L-*myc* probe. We thank Pål Møller, Eivind Hovig and Frances Jaques for their help and advice during preparation of this manuscript. This study has been supported by grants from the Norwegian Cancer Society. Toril Tefre is a fellow of The Norwegian Research Council for Science and the Humanities.

### References

ALT, F.W., DEPHINO, R., ZIMMERMAN, K. & 6 others (1986). The human *myc* gene family. *Cold. Spring Harbor Symp. Quant. Biol.*, **51**, 931.

ANTON-CULVER, H., CULVER, B.D., KUROSAKI, T., OSANN, K.E. & LEE, J.B. (1988). Incidence of lung cancer by histological type from a population-based registry. *Cancer Res.*, **48**, 6580.

BISHOP, J.M. (1987). The molecular genetics of cancer. *Science*, **235**, 305.

DEGREVE, J., BATTEY, J., FEDORKO. J. & 5 others (1988). The human L-*myc* gene encodes multiple nuclear phosphoproteins from alternatively processed mRNAs. *Mol. Cell. Biol.*, **8**, 4381.

FEINBERG, A.P. & VOGELSTEIN, B. (1983). A technique for radiolabelling DNA restriction endonuclease fragments for high specific activity. *Anal. Biochem.*, **132**, 6.

FRAUMENI, J.F. & BLOT, W.J. (1982). Lung and cancer epidemiology and prevention. In *Cancer Epidemiology and Prevention*, Scottenfield, D. & Freumeni, J.F. (eds) p. 564. W.B. Saunders: Philadelphia.

HEIGHWAY, J., TATCHER, N., CERNY, T. & HASLETON, P.S. (1986). Genetic predisposition to human lung cancer. *Br. J. Cancer*, **53**, 453.

IARC (1986). *Tobacco Smoking*, IARC monographs on the evaluation of the carcinogenic risk of chemicals to humans, Vol. 38, p. 221. Lyon.

IKEDA, I., ISHIZAKA, Y., OCHIAI, M. & 5 others (1988). No correlation between L-*myc* restriction fragment length polymorphism and malignancy of human colorectal cancers. *Jpn. J. Cancer Res.*, **79**, 674.

KAKEHI, Y. & YOSHIDA, O. (1989). Restriction fragment length polymorphism of the L-*myc* gene and susceptability to metastasis in renal cancer patients. *Int. J. Cancer*, **43**, 391.

KAWASHIMA, K., IMOTO, K., IZAWA, M. & 8 others (1987). Restriction fragment length polymorphism (RFLP) of L-*myc* is related to the progression of human colon and stomach cancers. *Proc. Jpn. Acad.*, **63**, 300.

KAWASHIMA, K., SHIKAMA, H., IMOTO, K. & 4 others (1988). Close correlation between restriction fragment length polymorphism of the L-*myc* gene and metastasis of human lung cancer to the lymph nodes and other organs. *Proc. Natl Acad. Sci. USA*, **85**, 2353.

KAYE, F., BATTEY, J., NAU, M. & 6 others (1988). Structure and expression of the human L-*myc* gene reveal a complex pattern of alternative mRNA processing. *Mol. Cell. Biol.*, **8**, 186.

KRYSTAL, G., BIRRER, M., WAY, J. & 5 others (1988). Multiple mechanisms for transcriptional regulation of the *myc* gene family in small cell lung cancer. *Mol. Cell. Biol.*, **8**, 3373.

KUNKEL, L.M., SMITH, K.D., BAYER, S.H. & 6 others (1977). Analysis of human Y-chromosome-specific reiterated DNA in chromosome variants. *Proc. Natl Acad. Sci. USA*, **74**, 1254.

KVÅLE, G. & JOHANSEN, G. (1982). Lung cancer in Norway (In Norwegian). *Tidskr. Nor. Lægeforen.*, **102**, 480.

NAU, M.M., BROOKS, B., BATTEY, J. & 7 others (1985). L-*myc*, a new *myc*-related gene amplified and expressed in human cell lung cancer. *Nature*, **318**, 69.

RYDBERG, D., TEFRE, T., ØVREBØ, S. & 6 others (1990). Ha-ras-1 alleles in Norwegian lung cancer patients. *Human Genet.* (in the press).

SAKSELA, K. (1987). Expression of the L-*myc* is under positive control by shortlived proteins. *Oncogene*, **1**, 291.

SOUTHERN, E.M. (1975). Detection of specific sequences among DNA fragments separated by gel electrophoresis. *J. Mol. Biol.*, **98**, 503.

ZIMMERMANN, K.A., YANCOPOULOS, G.D., COLLUM, R.G. & 9 others (1986). Differential expression of *myc* family genes during murine development. *Nature*, **319**, 780.